# Anti-Thermal Skin Aging Activity of Aqueous Extracts Derived from Apple Mint (*Mentha suaveolens *Ehrh.) in Human Dermal Fibroblasts

**DOI:** 10.1155/2018/4595982

**Published:** 2018-09-04

**Authors:** Dahee Son, Minkyung Kim, Hyunju Woo, Deokhoon Park, Eunsun Jung

**Affiliations:** Biospectrum Life Science Institute, A-1805, U-TOWER, 767, Sinsu-ro, Suji-gu, Yongin-si, Gyeonggi-do, 16827, Republic of Korea

## Abstract

Thermal skin aging refers to skin aging induced by heat shock treatment. Apple mint (*Mentha suaveolens *Ehrh.) has been used as a folk medicine to treat various diseases. However, the activity of apple mint in thermal skin aging has yet to be investigated. In this study, we conducted various biological assays to demonstrate the anti-thermal skin aging activity of extracts of apple mint leaves (ALE). As a result, ALE showed significant antioxidant activities and inhibited the production of reactive oxygen species (ROS), matrix metalloproteinases (MMPs), and interleukin-8 (IL-8) as well as suppressed mitogen-activated proteins kinases (MAPKs) such as extracellular signal regulated kinases (ERK), c-Jun N terminal kinases (JNK), and p38 MAPK triggered by heat shock treatment in human dermal fibroblasts (HDFs). Consequently, ALE could be used as attractive cosmetic materials with anti-thermal skin aging activity.

## 1. Introduction

Skin aging is a biological process, which leads to accumulation of structural and physiological alterations, in addition to changes in skin appearance [1]. It includes not only intrinsic aging that occurs naturally as getting older but also extrinsic aging triggered by a variety of environmental factors such as sun exposure and air pollutants induced by cigarette smoking and traffic dust [2–4].

Among the various environmental factors, heat is a crucial factor causing skin aging, which is called thermal skin aging [3]. Numerous studies have reported that heat causes the skin temperature to rise and affects skin aging through different ways. First, human skin is usually exposed to considerable amounts of infrared (IR) radiation from sunlight [5]. This IR radiation causes the generation of heat through molecular vibrations and rotations, which increases the skin temperature and has an influence in skin aging [6]. Also, human skin is frequently exposed to heat formed by using electronic devices such as smart phone, hair dryer, stove, and similar devices, which increase skin temperature and induce skin aging as well [3].

Matrix metalloproteinases (MMPs) are groups of zinc-containing proteinases, which are capable of degrading collagen and extracellular matrix (ECM) proteins consisting of skin dermal connective tissue [7]. Thus, increased MMPs play an important role in the development of skin aging. Previous studies have shown that heat shock stimulates the expression of MMPs. Heat shock has been reported to increase the levels of MMP-1 and MMP-3 in human dermal fibroblasts (HDFs) by the activation of extracellular signal regulated kinases (ERK) and c-Jun N terminal kinases (JNK). Additionally, it was shown that heat shock induces MMP-12 in human dermis* in vivo*, MMP-13 in a hairless mouse model, and the activation of p38 mitogen-activated protein kinases (MAPK) in HDFs [8–10].

The expression of MMPs is regulated by numerous signaling pathways. Among them, MMPs are controlled by the production of reactive oxygen species (ROS). ROS are known to be triggered by a number of stimuli and occurred in the natural process of aging. Recently, heat as an environmental factor was also reported to exert an influence on the production of ROS. According to previous studies, heat shock induced the generation of ROS and reduced the protein level of MAPK signaling in human keratinocytes, HaCaT cells [11]. Also, heat shock decreases the level of antioxidants such as glutathione (GSH), dehydrogenase 1 (NQO1), heme oxygenase (HO-1), and nuclear factor erythroid-derived 2-related factor 2 (Nrf2) regulating the oxidative response systems in HDFs [6].

As above, heat has been reported as a significant factor leading to skin aging. For this reason, it is necessary to study agents that can prevent thermal skin aging. In previous studies, (-)-epigallocatechin-3-gallate (EGCG) has shown to inhibit the expression of MMP-1 induced by heat shock in HDFs [12], and* Dunaliella salina *extracts suppress the expression of MMP-12 caused by heat shock in HaCaT cells [13]. However, there were a few studies about cosmetic raw materials with an inhibitory activity on thermal skin aging.

Apple mint (*Mentha suaveolens *Ehrh.) is an aromatic herb with sweet scent, which is usually found along streams and humid places [14]. It has been used in traditional medicines because of its diverse effects such as carminative, antispasmodic, diaphoretic, and analgesic [15]. It is also known to have biological efficacies such as antioxidant, anti-inflammatory, and antimicrobial [14, 16, 17]. Although numerous biological activities of apple mint have been demonstrated, its inhibitory activity against thermal skin aging has yet to be reported. Therefore, the aim of this study was to investigate the inhibitory effect of apple mint in skin aging induced by heat shock treatment and demonstrate its effectiveness as a raw material in cosmetics.

## 2. Materials and Methods

### 2.1. Materials and Reagent

The leaves of apple mint were purchased from Jeju Island (Korea). They were grown in the loam of Jeju Island for 1 year and collected during the period from May to July. 2 N Folin-Ciocalteu reagent, sodium carbonate, gallic acid, diethylene glycol, sodium hydroxide, rutin, 2, 2-diphenyl-1-picrylhydrazyl (DPPH), L-ascorbic acid, 2,2'-azino-bis(3-ethylbenzothiazoline-6-sulfonic acid) (ABTS), potassium persulfate, and ethanol were purchased from Sigma-Aldrich (St. Louis, MO, USA).

### 2.2. Preparation of Apple Mint Extracts

The leaves of apple mint were dried overnight in a hot air dryer at 50°C to pulverize into fine powders. 200 g of powders of apple mint leaves was extracted with 2 L of distilled water at 80°C. After filtering supernatants, the residues were freeze-dried to remove the remaining water and obtain final extracts of apple mint leaves (ALE).

### 2.3. Determination of Total Polyphenol Content

The total polyphenol content of ALE was determined by the modified previous method [18]. Briefly, ALE was mixed with of 2 N Folin-Ciocalteu reagent and 20% sodium carbonate solution. After incubating this mixture for 30 min, the absorbance was measured at 725 nm using Gen5™ UV-Vis spectrophotometer (BioTek, Winooski, VT, USA). Gallic acid was used as a standard to calculate the total polyphenol content.

### 2.4. Determination of Total Flavonoid Content

The total flavonoid content of ALE was determined by the modified previous method [19]. Briefly, ALE was mixed with 90% diethylene glycol and 1N sodium hydroxide solution. After incubating this mixture for 30 min, the absorbance was measured at 420 nm using a spectrophotometer. Rutin was used as a standard to calculate the total flavonoid content.

### 2.5. Measurement of DPPH Radical Scavenging Activity

The DPPH radical scavenging activity of ALE was determined by the modified previous method [20]. In brief, ALE was mixed with 200 *μ*M DPPH reagent in equal amounts. After incubating this mixture for 30 min, the absorbance was measured at 517 nm using a spectrophotometer. DPPH radical scavenging activity was calculated by the following formula, % scavenging activity = [(A_control_ − A_sample_)/A_control_ × 100]. A_control_ and A_sample_ indicate the absorbance of only DPPH reagent and ALE-treated groups, respectively. L-ascorbic acid was used as a positive control with antioxidant activity.

### 2.6. Measurement of ABTS Radical Scavenging Activity

The ABTS radical scavenging activity of ALE was determined by the modified previous method [21]. The ABTS radical solution was prepared by incubating 7 mM ABTS solution with 2.46 mM potassium persulfate solution overnight. This ABTS radical solution was diluted in ethanol to obtain an absorbance of 0.7 units at 734 nm by a spectrophotometer. ALE reacted with ABTS radical solution for 30 min. Then, the absorbance was measured at 734 nm using a spectrophotometer. ABTS radical scavenging activity was calculated by the following formula, % scavenging activity = [(A_control_  −  A_sample_)/A_control_  ×  100]. A_control_ and A_sample_ indicate the absorbance of only ABTS reagent and ALE-treated group, respectively. L-ascorbic acid was used as a positive control.

### 2.7. Cell Culture

Human dermal fibroblasts (HDFs) were purchased from American Type Culture Collection (ATCC; Manassas, VA, USA). HDFs were cultured in Dulbecco's modified Eagle's medium (DMEM; Welgene, Daegu, Korea) supplemented with 10% fetal bovine serum (FBS; Welgene) and 1% (v/v) of penicillin-streptomycin (Welgene) in a 5% CO_2_ humidified incubator at 37°C. All experiments were carried out when HDFs were grown to 70-80% confluence in 60 mm^2^ dishes.

### 2.8. Heat Treatment

For heat shock treatment, HDFs cultured dishes were submerged in a circulating water bath thermoregulated at 37°C (control) or 45°C (heat shock treatment) for 30 min. After heating, culture media were changed with fresh media and incubated for the indicated times to stimulate heat stress in HDFs. When required, HDFs were pretreated with ALE for 24 hr before heat shock treatment and incubated for indicated times.

### 2.9. Measurement of Intracellular ROS Level

Intracellular ROS level was detected by using 2', 7'-dichlorofluorescin diacetate (DCF-DA; Thermo Scientific, Carlsbad, CA, USA) [22]. HDFs were pretreated with various concentrations of ALE from 10 to 100 *μ*g/mL for 24 hr, followed by heat shock treatment. Then, HDFs were treated immediately with 50 *μ*M DCF-DA for 30 min. The fluorescence intensity was measured at a specific excitation/emission wavelength (485/535 nm) using Infinite^®^ F200 PRO (Tecan, AG, Mannedorf, Switzerland). HDFs stained with DCF-DA were imaged by an EVOS fluorescent microscope (Advanced Microscopy Group, Bothell, WA, USA). Also, the ROS level was detected by a FACSCalibur flow cytometry (Becton Dickinson, San Jose, CA, USA) [23].

### 2.10. Quantitative Real-Time Polymerase Chain Reaction (qRT-PCR) Analysis

The mRNA expression of MMPs was determined by qRT-PCR. HDFs were treated with various concentrations of ALE from 10 to 100 *μ*g/mL for 24 hr after heat shock treatment. Total RNA of HDFs was isolated using the TRIzol reagent (Invitrogen, Carlsbad, CA, USA). 1 *μ*g of RNA was reverse transcribed to cDNA using amfiRivert Platinum cDNA synthesis Master Mix kit (GenDEPOT, Barker, TX, USA). The synthesized cDNA was amplified by ABI PRISM 7300 System (Applied Biosystems, Foster City, CA, USA) using PowerUp SYBR green Master Mix (Applied Biosystems) and primers (Qiagen, Valencia, CA, USA). The following conditions of PCR cycling were used: initiation at 50°C for 2 min and 95°C for 10 min, followed by 40 cycles of 95°C for 15 s and 60°C for 2 min. The relative expression of each gene was calculated by Ct analysis. Glyceraldehyde-3-phosphate dehydrogenase (GAPDH) was used as an internal control for normalization. Each procedure was performed according to the manufacturer's instructions.

### 2.11. Enzyme-Linked Immunosorbent Assay (ELISA)

The level of interlukin-8 (IL-8) in the cell supernatants was measured by Human IL-8/CXCL8 Quantikine ELISA Kit (R&D system Inc., Minneapolis, MN, USA) according to the manufacturer's protocol. HDFs were treated with various concentrations of ALE from 10 to 100 *μ*g/mL for 48 hr after heat shock treatment and cell supernatants were collected after ALE treatment.

### 2.12. Western Blot Analysis

The level of protein in the total and phosphorylated form of ERK, JNK, and p38 was detected by western blot analysis. HDFs were pretreated with various concentrations of ALE from 10 to 100 *μ*g/mL for 24 hr, followed by heat shock treatment. Subsequently, HDFs were lysed in radioimmunoprecipitation assay buffer (RIPA; Thermo Scientific) containing Halt Protease and Phosphatase Inhibitor Cocktail (Thermo Scientific). Protein concentrations of the cell lysates were measured by a Pierce BCA protein assay kit (Thermo Scientific). Cell lysates were loaded on 10% Bis-Tris Gel (Invitrogen) and transferred to a nitrocellulose membrane using an iBlot transfer system (Invitrogen). The membranes were blocked with 5% skim milk (BD Biosciences, Franklin, NJ, USA) in tris-buffered saline containing 0.1% v/v Tween-20 for 1 hr. The blocked membranes were incubated with primary antibody (diluted 1:1000, Cell signaling, Beverly, MA, USA) overnight, followed by incubation with secondary antibody (diluted 1:1000, Santa Cruz Biotechnology Inc., CA, USA) conjugated with horseradish peroxidase (HRP) for 1 hr. The immunoreactive bands were detected by using a PicoEPD Western Reagent (ElpisBiotech, Daejeon, Korea) and visualized by Image Quant LAS 500 (GE Healthcare Life Sciences, Shanghai, China).

### 2.13. Statistical Analysis

All data were expressed as mean ± standard deviation of independent experiments in triplicate. Statistical significance of data was determined by Student's* t*-test. ^*∗*^*p*<0.05 and ^*∗∗*^*p*<0.01 was considered to be significant.

## 3. Results and Discussion

### 3.1. Result

#### 3.1.1. Total Polyphenol, Total Flavonoid Content, and Antioxidant Activity of ALE

Total polyphenol content, total flavonoid content, DPPH radical scavenging activity, and ABTS radical scavenging activity assay were performed to measure the antioxidant activity of ALE. As shown in Table 1, the total polyphenol content of ALE was 103.7 ± 0.9 *μ*g GAE/mg DM and the total flavonoid content of ALE was 7.1 ± 0.1 *μ*g RE/mg DM. ALE also inhibited 50% of DPPH and ABTS radicals at the concentration of 86.0 ± 6.2 *μ*g DM/mL and 58.5 ± 0.5 *μ*g DM/mL, respectively. L-ascorbic acid, used as a positive control, inhibited 50% of DPPH and ABTS radicals at the concentration of 8.3 ± 0.5 *μ*g DM/mL and 10.1 ± 0.1 *μ*g DM/mL, respectively.

#### 3.1.2. ALE Inhibits the Intracellular ROS Generation Induced by Heat Shock Treatment in HDFs

**Figure 1:**
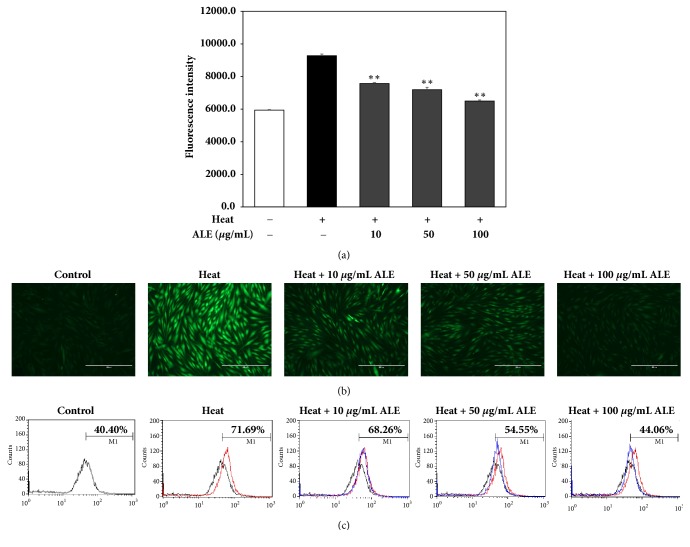
Inhibitory effect of ALE against ROS generation induced by heat shock treatment in HDFs. (a) ROS level was measured by fluorescence intensity in the wavelength of excitation 485 nm and emission 535 nm. (b) Fluorescence of HDFs induced by ROS was imaged. (c) ROS level was also measured by flow cytometry. The results were expressed as the means ± standard deviation (*n*=3). ^*∗∗*^*p*<0.01 vs. heat-treated group.

The level of intracellular ROS was determined using DCF-DA. The inhibitory activity of ALE against ROS formation induced by heat shock treatment was shown in Figure 1. The fluorescence intensity of the heat-treated group was increased by 56.2% compared with the control group. On the other hand, the fluorescence intensity of the ALE-treated groups was significantly decreased by 28.8, 35.2, and 46.8% at concentrations of 10, 50, and 100 *μ*g/mL, respectively, compared with heat-treated group (Figure 1(a)). These differences of fluorescence intensity were observed in the image of HDFs stained with DCF-DA (Figure 1(b)). Also, the intracellular ROS level was detected by flow cytometry. The range of M1 indicates the percentage of increased fluorescence in each subpopulation of the cells. The ROS level of the heat-treated group was increased by 71.7% compared with the control group, 40.4%. However, the ROS levels of ALE-treated groups were decreased by 68.3, 54.6, and 44.1% at concentrations of 10, 50, and 100 *μ*g/mL, respectively (Figure 1(c)).

#### 3.1.3. ALE Prevents the Expression of MMPs Induced by Heat Shock Treatment in HDFs

**Figure 2:**
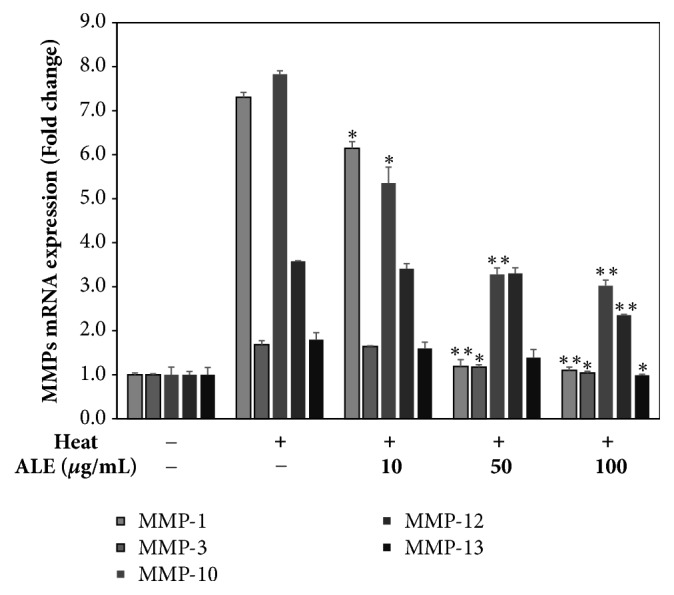
Inhibitory effect of ALE against the expression of MMPs induced by heat shock treatment in HDFs. The mRNA level of MMP-1, MMP-3, MMP-10, MMP-12, and MMP-13 was measured by qRT-PCR. The results were expressed as the means ± standard deviation (*n*=3). ^*∗*^*p*<0.05 and ^*∗∗*^*p*<0.01 vs. heat-treated group.

The mRNA level of MMPs was measured using qRT-PCR. Each group was expressed as the fold value based on the control group and these results were shown in Figure 2. The mRNA levels of MMP-1, MMP-3, MMP-10, MMP-12, and MMP-13 induced by heat shock treatment were increased to 7.3 ± 0.1, 1.7 ± 0.1, 7.8 ± 0.1, 3.6 ± 0.0, and 1.8 ± 0.2 fold compared with the control group, respectively. On the other hand, ALE significantly inhibited the expression of MMP-1, MMP-3, MMP-10, MMP-12, and MMP-13 by 1.1 ± 0.1, 1.0 ± 0.0, 3.0 ± 0.1, 2.4 ± 0.0, and 1.0 ± 0.0 fold compared with the heat-treated group at 100 *μ*g/mL, respectively.

#### 3.1.4. ALE Suppresses the Activation of Phosphorylated-ERK (p-ERK), Phosphorylated-JNK (p-JNK) and Phosphorylated-p38 (p-p38) Induced by Heat Shock Treatment in HDFs

**Figure 3:**
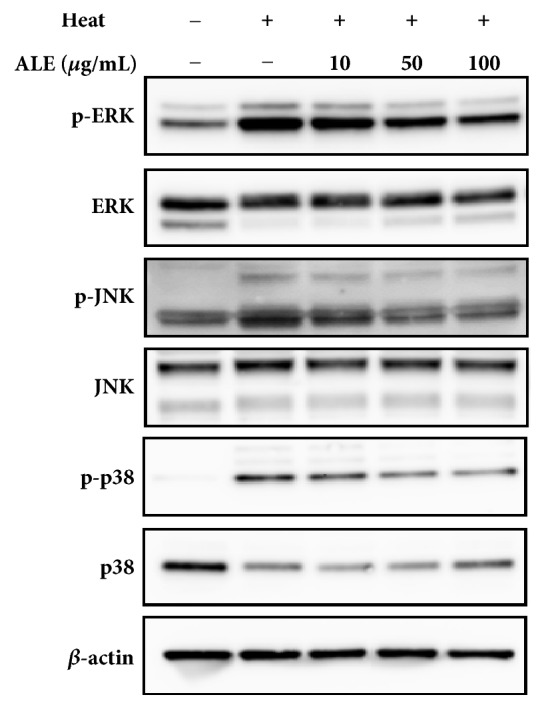
Inhibitory effect of ALE against the expression of ERK, JNK, and p38 induced by heat shock treatment in HDFs. The protein level of ERK, phospho-ERK (p-ERK), JNK, phospho-JNK (p-JNK), p38, and phospho-p38 (p-p38) was determined by western blot. Each of the figures was representative of three independent experiments. Beta-actin (*β*-actin) was used as an internal control to confirm equal protein loading.

The total and phosphorylated forms of ERK, JNK, and p38 were analyzed by western blot assay. As shown in Figure 3, the group treated with only heat shock resulted in the activation of p-ERK, p-JNK, and p-p38, while ALE showed an inhibitory activity of p-ERK, p-JNK, and p-p38 in a dose-dependent manner.

#### 3.1.5. ALE Prevents the Formation of Interlukin-8 (IL-8) Induced by Heat Shock Treatment in HDFs

**Figure 4:**
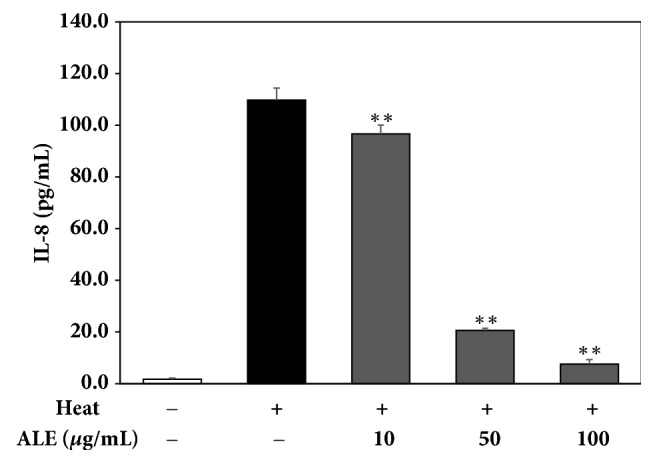
Inhibitory effect of ALE against the expression of interlukin-8 (IL-8) induced by heat shock treatment in HDFs. The content of IL-8 was determined by ELISA. The results were expressed as the means ± standard deviation (*n*=3). ^*∗∗*^*p*<0.01 vs. heat-treated group.

The inhibitory activity of ALE against the expression of IL-8 induced by heat shock treatment was measured by ELISA. As shown in Figure 4, ALE significantly inhibited the formation of IL-8 in a dose-dependent manner. The concentration of IL-8 in the heat-treated group was 109.8 ± 4.6 pg/mL, while the concentrations of IL-8 in ALE-treated groups were 96.7 ± 3.4, 20.6 ± 0.8, and 7.5 ± 1.7 pg/mL at 10, 50, and 100 ppm, respectively.

### 3.2. Discussion

Heat as an environmental factor plays an important key role in skin aging. Numerous studies have reported that heat was causative of increase in MMPs and ROS generation and decrease in the antioxidant activity, which are relevant to skin aging [6, 8–11]. Therefore, many studies are required to find out agents with an inhibitory effect against thermal skin aging.

Apple mint is an attractive herb used as folk medicines to cure several diseases [14–17]. However, its anti-thermal aging activity has yet to be reported. Therefore, we carried out biological experiments to confirm the activity of apple mint against thermal skin aging stimulated by heat shock treatment in HDFs.

A number of studies have presented that ROS and other environmental factors increase the level of MMPs by activating MAPKs signaling [24–26]. Among the various factors, heat shock was also demonstrated that it induces the generation of ROS and plays a crucial role in the regulation of MMPs through MAPKs signaling [11]. Thus, antioxidant activity is important because of this ability to eliminate ROS, which is the main cause of skin aging. Previous studies have shown that extracts with antioxidant activity could prevent the induction of ROS by various stimuli.* Ixora Parviflora *extract, which exhibited antioxidant activities inhibited the production of ROS induced by ultraviolet (UV) radiation in HDFs [27]. We found that ALE was effective in preventing the formation of ROS stimulated by heat shock treatment (Figure 1). This efficacy could be predicted because of its high polyphenolic compounds and free radicals scavenging activities (Table 1).

MMPs regulated by ROS are also known to accelerate skin aging by promoting the degradation of ECM, including type I collagen, elastin, and fibronectin [28]. Heat shock has been reported to influence the upregulation of collagenases (MMP-1, MMP-13), gelatinases (MMP-9), stromelysins (MMP-3), and metalloelastase (MMP-12) in* in vitro *and* in vivo *models [8–11]. We observed that heat shock induced the expression of MMP-1, MMP-3, MMP-10, MMP-12, and MMP-13 in HDFs and ALE suppressed MMPs expression induced by heat shock treatment (Figure 2).

There are three types of MAPKs such as ERK, JNK, and p38. These MAPKs are known to play an essential role in the expression of activator protein-1 (AP-1) activity, which plays an important role in the transcriptional regulation of MMPs [28, 29]. In a previous study, it was reported that heat shock induced the expression of MMP-1 and MMP-3 through ERK and JNK activation in HaCaT cells [8]. We found that heat shock induced phosphorylated form of ERK, JNK, and p38 in HDFs and ALE prevented the activation of these MAPKs signaling induced by heat shock treatment (Figure 3). Thus, ALE could be predicted to inhibit MMP expression by preventing the activation of ERK, JNK and p38.

Interleukin-8 (IL-8) is a cytokine that is involved in the UV-induced skin inflammation and diseases [30, 31]. In a recent study, the production of IL-8 was stimulated by IR radiation in HDFs. We found that heat shock induced the production of IL-8 and ALE inhibited the release of IL-8 in a dose-dependent manner (Figure 4). Thus, this result presents the fact that heat shock may be involved in skin inflammation. However, further studies are needed to explain how heat shock stimulates IL-8 expression and how ALE inhibited IL-8 production.

Consequently, it is expected that ALE with antioxidant activities, suppressing ROS generation, reducing MMPs expression, and inhibiting MAPKs activation could be useful as cosmetic agents with anti-thermal skin aging activity.

## 4. Conclusions

We demonstrated that ALE has significant antioxidant activities and prevents the formation of ROS, MMPs, IL-8, and MAPK signaling induced by heat shock treatment. In conclusion, our findings suggest that ALE is an attractive and potential agent with anti-thermal skin aging activity, which could be available to cosmetic industry.

## Figures and Tables

**Table 1:** Total polyphenol, total flavonoid content, and antioxidant activity of ALE. Total polyphenol content was expressed as gallic acid equivalents (GAE) and total flavonoid content was expressed as rutin equivalents (RE). Antioxidant activities were expressed as DPPH-scavenging activity (DPPH·SC_50_) and ABTS-scavenging activity (ABTS·SC_50_). DM refers to dried materials and SC_50_ indicates the concentration of ALE required to reduce 50% DPPH and ABTS radicals. The results were expressed as the mean ± standard deviation (*n*=3).

	**Total Polyphenol**	**Total Flavonoid**	**DPPH·SC**_**50**_	**ABTS·SC**_**50**_
	**(** **µ** **g GAE/mg DM)**	**(** **µ** **g RE/mg DM)**	**(** **µ** **g DM/mL)**	**(** **µ** **g DM/mL)**
ALE	103.7 ± 0.9	7.1 ± 0.1	86.0 ± 6.2	58.5 ± 0.5
L-ascorbic acid	-	-	8.3 ± 0.5	10.1 ± 0.1

## Data Availability

No data were used to support this study.
